# Assessing key soft skills in organizational contexts: development and validation of the multiple soft skills assessment tool

**DOI:** 10.3389/fpsyg.2024.1405822

**Published:** 2024-10-25

**Authors:** Daiana Colledani, Egidio Robusto, Pasquale Anselmi

**Affiliations:** ^1^Department of Philosophy, Sociology, Education and Applied Psychology, University of Padua, Padua, Italy; ^2^Department of Psychology, Sapienza University of Rome, Rome, Italy

**Keywords:** soft skills, performance, job satisfaction, burnout, network analysis, measurement invariance, item response theory

## Abstract

**Introduction:**

Soft skills, also known as transversal skills, have gained significant attention in the organizational context due to their positive impact on various work-related outcomes. The present study aimed to develop and validate the Multiple Soft Skills Assessment Tool (MSSAT), a short self-report instrument that evaluates interpersonal skills (initiative-resourcefulness, assertiveness, conflict management), interpersonal communication skills, decision-making style (adaptive and maladaptive), and moral integrity.

**Methods:**

The scale development process involved selecting and adapting relevant items from existing scales and employing a cross-validation approach with a large sample of workers from diverse organizational settings and job positions (*N* = 639). In the first step, 28 items were carefully chosen from an item pool of 64 items based on their content, factor loadings, item response theory analyses, differential item functioning, and fit statistics. Next, the structure of the resulting scale was evaluated through confirmatory factor analyses.

**Results:**

The MSSAT demonstrated gender invariance and good reliability and validity. The results of a network analysis confirmed the relationships between soft skills and positive work-related outcomes. Notably, interpersonal communication skills and moral integrity emerged as crucial skills.

**Discussion:**

The MSSAT is a valuable tool for organizations to assess the soft skills of their employees, thereby contributing to design targeted development programs.

## Introduction

Soft skills, often referred to as transversal, non-technical or social skills, comprise a wide range of personal qualities, behaviors, and competencies that go beyond technical expertise. Yorke (2006) characterizes them as a blend of dispositions, understandings, attributes, and practices. Their multifaceted nature is reflected in the literature, which abounds with models and taxonomies delineating various soft skills. These encompass a wide spectrum of abilities, such as conflict resolution, decision-making, presentation skills, teamwork, communication skills, relationship management, leadership, adaptability, problem-solving, ethics, and values (e.g., Cimatti, 2016; Khaouja et al., 2019; Soto et al., 2022; Verma and Bedi, 2008).

While labor market studies have traditionally focused on technical skills and knowledge, currently there is a growing recognition of the importance of soft skills (Balcar, 2016; Ciappei and Cinque, 2014; Eshet, 2004; Seligman, 2002). This shift in focus stems from a deeper understanding of the positive impact of soft skills on successful careers and employability (Charoensap-Kelly et al., 2016; Salleh et al., 2010; Sharma, 2018; Styron, 2023; Yahyazadeh-Jeloudar and Lotfi-Goodarzi, 2012). A large body of research suggests that employees with strong soft skills not only improve their job performance (Ibrahim et al., 2017), but are also less prone to poor psychophysical health and burnout (Rosa and Madonna, 2020; Semaan et al., 2021). In addition, soft skills were found to increase individual drive and passion, which promotes overall productivity and organizational growth (Murugan and Sujatha, 2020; Nugraha et al., 2021).

Given the recognized contribution of soft skills in the promotion of successful careers, their role in enhancing employability, and their potential for improvement through appropriate training programs, soft skills have become a topic of great interest to human resource professionals, and their assessment has become a standard practice in personnel selection and training design (Charoensap-Kelly et al., 2016; Gibb, 2014; Salleh et al., 2010; Sharma, 2018; Styron, 2023).

Having available a tool that reliably and effectively measures key soft skills in different organizational contexts would be of great value in a number of ways. From an applied perspective, it could facilitate recruitment and selection processes by enabling the effective and efficient assessment of soft skills required for success in candidates (Asefer and Abidin, 2021; Nickson et al., 2012). This is particularly relevant in modern times, as the labor market is dynamic and constantly seeking individuals with the employability skills required by workplaces. However, the literature reports a significant gap between the soft skills desired by employers and the level of these skills among candidates and new hires, often resulting in many positions remaining vacant (Abelha et al., 2020; Hurrell, 2016; Jackson and Bridgstock, 2018; Nisha and Rajasekaran, 2018). The skills gap is a recognized talent management challenge (McDonnell, 2011). In addition, a scale that efficiently assesses key soft skills would also facilitate ongoing monitoring providing valuable information on employees’ strengths and areas for improvement, which would be useful in designing targeted training and development programs that foster both personal and professional growth (Adhvaryu et al., 2023; Widad and Abdellah, 2022). Using a scale that efficiently assesses soft skills within the organization can also be useful in inspiring initiatives to improve organizational culture, including diversity and inclusion efforts, conflict resolution, and employee well-being programs (Juhász et al., 2023). Moreover, understanding the soft skills profiles of team members can help managers assemble balanced teams with complementary strengths, thereby fostering better collaboration, innovation, and problem solving. Finally, from a broader perspective, a scale that reliably assesses key soft skills in different organizational contexts can facilitate their study in real-world settings, leading to a more nuanced understanding of how they interact and affect organizational contexts. Moreover, it would promote the study of the transferability of these competencies across contexts, roles and sectors, helping to identify which soft skills have value universally and which are context-specific. In fact, although the soft skills required for different job profiles vary to some extent, some core soft skills are considered essential in most contemporary business environments and sectors (Alsabbah and Ibrahim, 2013; Kyllonen, 2013; Paddi, 2014).

This work aims to develop and validate the Multiple Soft Skills Assessment Tool (MSSAT), a short self-report instrument designed to efficiently and reliably measure key soft skills in different organizational contexts. MSSAT focuses on four relevant soft skills domains, namely interpersonal skills, communication skills, decision-making style, and moral integrity. These skills were chosen because they are widely accepted in different taxonomies and are considered important in different professional positions (Khaouja et al., 2019; Soto et al., 2022). The decision to limit the number of items for each skill was driven by the practical need of organizations to obtain quick and accurate assessments. In fact, short assessment tools are valuable to organizations because they facilitate accurate responses and minimize the time required to complete the assessment (Burisch, 1984; Fisher et al., 2016; Sharma, 2022). MSSAT is expected to be a useful tool for applications in the aforementioned contexts.

The development and validation of the MSSAT closely adhere to best practices in the literature (Boateng et al., 2018; Hinkin, 1998). First, the dimensions of interest are identified and an initial pool of items is constructed to assess them. Next, the item pool is administered to an appropriate sample of individuals, and the items to be included in the scale are selected. Finally, the psychometric properties of the scale, including dimensionality, reliability, validity, and nomological network, are assessed. Psychometrically sound measures (e.g., Colledani et al., 2024; Hinkin and Schriesheim, 1989; Kumar and Beyerlein, 1991; Tassé et al., 2016) have been obtained using these practices.

Section “Soft Skills of Interest and Item Pool” describes the identification of the soft skills of interest and the construction of an item pool from which the items were selected to develop the MSSAT. Section “Method” describes the procedures employed to select the items and to validate the scale.

## Soft skills of interest and item pool

To develop the MSSAT, the soft skills of interest were identified and an item pool was constructed, consisting of items selected from instruments available in the literature. This method of test development is described, for instance, in Boateng et al. (2018) and Hinkin (1995). The items were drawn from instruments not specifically designed for the organizational context and were carefully reformulated by the authors of this study (experts in the fields of organizational psychology and psychometrics) to ensure their suitability for effectively assessing soft skills in organizational settings.

### Interpersonal skills

Three interpersonal skills were considered in the development of the MSSAT because of their profound impact on professional success: initiative-resourcefulness, assertiveness, and conflict management. Initiative-resourcefulness denotes the ability to engage with new and interesting people, as well as the ability to present oneself appropriately. This skill is crucial in organizational settings and is often associated with greater job satisfaction and better performance (Agba, 2018; Akla and Indradewa, 2022; Nadim et al., 2012). Assertiveness denotes the ability to pursue one’s rights, will, and needs in a firm but non-aggressive manner. It is a crucial skill that can reduce conflict and decrease stress, burnout, and turnover intentions (Butt and Zahid, 2015; Ellis and Miller, 1993; Tănase et al., 2012). Finally, conflict management denotes the ability to prevent interpersonal conflict and manage conflict situations effectively. Consistent with the literature, conflict is an inevitable occurrence in organizations and often results in reduced employee and organizational flourishing (Awan and Saeed, 2015; Henry, 2009). However, when properly managed, conflict can be used to drive change and improve employee satisfaction and organizational performance.

To construct the subscales measuring these three interpersonal skills, 22 items were extracted from three of the five subscales included in the Interpersonal Competence Questionnaire (Buhrmester et al., 1988), namely from Initiating Relationships, Expressing Displeasure with Others’ Actions, and Managing Interpersonal Conflicts. Since the items were originally developed to assess these interpersonal skills in the context of peer relationships, they were reformulated for use in the organizational context.

### Communication skills

Communication is the process of exchanging information to reach a common understanding. Effective interpersonal communication skills are critical to achieving organizational goals and fostering professional success. Research has consistently demonstrated the critical role of interpersonal communication skills in boosting job and team performance (Dehghan and Ma’toufi, 2016; Keerativutisest and Hanson, 2017). Furthermore, research has shown that good communication skills increase organizational commitment and buffer the onset of emotional exhaustion while promoting self-actualization (Bambacas and Patrickson, 2008; Emold et al., 2011; Paksoy et al., 2017).

To construct the scale measuring communication skills, nine items were selected from the Interpersonal Communication Satisfaction Inventory (Hecht, 1978). This 19-item scale measures satisfaction with interpersonal communication by assessing respondents’ reactions to recent conversations in which they have been involved. Although the scale does not directly assess communication skills, it does include several items that refer to functional communication behaviors. In addition, since communication satisfaction is often associated with competent communication, it may serve as an indirect indicator of communication competence (Hecht, 1978). The nine items selected for inclusion in the MSSAT were chosen because they were considered most appropriate for assessing communication skills in a professional context.

### Decision-making

Extensive research has identified a strong relationship between poor decision-making skills and detrimental employee outcomes, including increased risk-taking, maladaptive coping mechanisms, perceived stress, emotional exhaustion, depersonalization, and sleep disturbance (Allwood and Salo, 2012; Del Missier et al., 2012; Valieva, 2020; Weller et al., 2012). In contrast, robust decision-making skills have been found to enable the formulation of adaptive goals and the adoption of appropriate actions to achieve them (Byrnes, 2013; Byrnes et al., 1999), and have been consistently recognized as a critical factor in organizational effectiveness, employee engagement, work commitment, and job satisfaction (Ceschi et al., 2017).

The 22 items of the Melbourne Decision-making Questionnaire (DMQ; Mann et al., 1997) were used to construct the decision-making style measure to be included in the MSSAT. The DMQ is a well-known instrument that assesses decision-making in terms of four primary styles: vigilance, hypervigilance, buck-passing, and procrastination. Vigilance involves a series of processes aimed at clarifying goals, seeking information, exploring and evaluating alternatives, and ultimately making a decision. Vigilance is considered the only decision-making style that allows for functional and rational choices (Mann et al., 1997). In contrast, hypervigilance involves a frantic search for solutions, often driven by a sense of time pressure. Decision makers in this condition tend to impulsively adopt hastily devised solutions, usually aimed at providing immediate relief. Hypervigilance is characterized by a heightened emotional state, which leads to a disregard for the full range of consequences. Buck-passing is the act of shifting the responsibility for making a decision to someone else. This behavior refers to the act of avoiding personal responsibility and shifting the buck to others. Additionally, procrastination involves replacing high-priority tasks with lower-priority activities and engaging in pleasurable distractions. This behavior can lead to the postponement or avoidance of important decisions.

The decision-making measure of the MSSAT was constructed using all the DMQ items because of their generic wording, which makes them applicable to different contexts (including the workplace), and the brevity of the subscales (each containing 5 or 6 items).

### Integrity

Integrity has received considerable attention in the literature on personnel selection in recent decades (Schmidt and Hunter, 1998). Early studies focused mainly on the positive impact of ethical leadership (De Carlo et al., 2020; Engelbrecht et al., 2017). However, subsequent research has also established the positive role of employee integrity. For instance, employee integrity has been shown to be positively correlated with improved performance (Inwald et al., 1991; Luther, 2000; Ones et al., 1993; Posthuma and Maertz, 2003) and negatively associated with counterproductive work behaviors (Van Iddekinge et al., 2012).

To assess integrity, 12 items were selected from the 18-item Integrity Scale developed by Schlenker (2008). This scale measures the value placed on ethical behavior, adherence to principles despite temptation or personal cost, and refusal to justify unethical behavior, and includes facets of integrity such as truthfulness and honesty. Higher scores indicate greater commitment to ethical principles and higher levels of integrity (Schlenker, 2008). The 12 items considered in the development of the MSSAT measure of integrity are those most appropriate for assessing integrity in organizational contexts.

The resulting item pool consists of 64 items that were carefully selected by the authors of the present study based on their relevance to the organizational contexts, and in some cases reformulated to optimize their appropriateness to work contexts. This item pool constitutes the basis for the construction of the MSSAT.

## Method

### Participants

A total of 639 participants (mean age = 38.08; *SD* = 1.45; males = 352, 55.1%) were recruited through snowball sampling procedure. The majority of them were white-collar workers (*N* = 269, 42.1%), followed by blue-collar workers (*N* = 90, 14.1%). Other occupations included shop assistants (*N* = 26, 4.1%), health professionals (*N* = 73, 11.4%), and teachers and educators (*N* = 71, 11.1%). The remaining 17.2% (*N* = 110) comprised managers, professionals, freelancers, and entrepreneurs from various sectors. The majority of participants worked full-time (*N* = 346, 75.87%, over 30 h per week) and had a high level of seniority (*N* = 249, 39.97%, up 10 years; *N* = 390, 61.03%, over 10 years).

Participants did not receive any compensation for their involvement in the study, and the only inclusion criterion was being a worker aged 18 years or above. Prior to access to the online survey, participants were asked to provide electronic informed consent, which explained the purpose of the study, the estimated duration of the task, and the option to withdraw consent at any time during the study. Participation in the study was anonymous and voluntary. The study was conducted in adherence to the ethical principles for research outlined in the Declaration of Helsinki and approved by the local committee for psychological research of the University of Padua.

### Materials and procedure

All participants were presented with an online survey consisting of a series of closed-ended questions regarding their demographics (e.g., including age, gender, professional sector, and contract type), the 67 items of the item pool, and three additional scales measuring burnout job satisfaction, and performance.

Job satisfaction was evaluated using the scale developed by Dazzi et al. (1998). The scale comprises six items (e.g., “I feel satisfied with my work”) with higher scores indicating greater satisfaction with one’s work (Cronbach’s *α* = 0.82 in the current sample). Burnout was measured using the Qu-Bo test developed by De Carlo et al. (2008/2011). The instrument consists of nine items, divided into three subdimensions of three items each: exhaustion (e.g., “I feel burned out from my work”), cynicism (e.g., “My work has no importance”), and reduced sense of personal accomplishment (e.g., “I feel incapable of doing my job”). In this study, a total scale score was computed, with higher scores indicating higher levels of burnout (Cronbach’s *α* = 0.84 in the current sample).

The items pertaining soft-skills, job satisfaction, and burnout employed a 4-point response scale (1 = “*Completely disagree*” to 4 = “*Completely agree*”).

Finally, job performance was evaluated using four items. Two of them (e.g., “How would you rate your job performance in the past year?”; “Over the past year, how did your supervisors and/or co-workers rate your job performance?”) used a 10-point Likert scale format (1 = “*Very poor performance*”) to 10 (“*Very good performance*”), while the other two items (e.g., “Please indicate the percentage of your work goals achieved during the past year”; “Please indicate the percentage to which your manager and/or co-workers believe you have been successful in achieving your work goals over the past year”) asked participants to express their work goal achievement as a percentage from 10% (“*Very poor performance*”) to 100% (“*Very good performance*”; in the current sample, Cronbach’s *α* = 0.86).

### Data analysis

A two-step approach was used to construct and validate the MSSAT. In the first step, the data sample was divided into two parts. The first part (calibration dataset; *N* = 319) was analyzed to identify the best items, four for each dimension, for inclusion in the MSSAT, while the second part (validation dataset; *N* = 320) was used to confirm the factorial structure of the scale. In the second step, the entire dataset (*N* = 639) was analyzed to examine the measurement invariance of the instrument across gender, as well as its reliability, validity, and nomological network. The sizes of the calibration and validation datasets, as well as the size of the entire dataset, are appropriate according to the common criteria of at least four participants for each item (Hinkin, 1998; Rummel, 1970) and at least 300 participants overall (Boateng et al., 2018; Comrey, 1988; Guadagnoli and Velicer, 1988).

#### Development of the MSSAT

To select the most appropriate items for inclusion in the MSSAT, a subsample comprising responses from 319 participants was used (mean age = 38.60 years, *SD* = 14.39; males = 128, 40.1%). The subsample was obtained by randomly dividing the entire dataset into two parts. The items related to the four soft skills domains, namely interpersonal skills (initiative-resourcefulness, assertiveness, conflict management), interpersonal communication skills, decision-making style (vigilance, hypervigilance, procrastination, and buck-passing), and integrity, were analyzed separately using the following procedure. First, the minimum average partial (MAP; Velicer, 1976) test was used to evaluate the dimensionality of each scale. The data of each scale were then analyzed using factor analysis and item response theory (IRT). In particular, exploratory factor analysis (EFA) and the graded response model (GRM; Samejima, 1969) were applied. These methods are commonly used in scale development (Colledani et al., 2018a; Colledani et al., 2019a, 2019b; Colledani et al., 2018b; Lalor et al., 2016; Tassé et al., 2016; Zanon et al., 2016) and provide a convenient framework for evaluating numerous relevant attributes of the items, such as location on the latent trait continuum, discriminability, item fit, and differential item functioning (DIF).

In this work, item fit was evaluated using the signed chi-square test (S-χ^2^; Orlando and Thissen, 2000). A significant *p-*value for the S-χ^2^ of an item indicates that the responses to the item have a poor fit to the IRT model, a condition referred to as misfit. Gender DIF was assessed through ordinal logistic regression analyses. This approach uses the estimates of an IRT model (the GRM in this study) to quantify the trait levels of participants (i.e., their level in each of the soft skills under consideration) and a dichotomous variable to indicate their group membership (i.e., male *vs* female). To identify uniform DIF, only the impact of trait levels and group membership (gender group) on the item responses was considered, while to identify nonuniform DIF, the interaction term between trait levels and group membership was also considered. For items exhibiting DIF (uniform, nonuniform, or both), the McFadden Pseudo-*R*^2^ was used to determine the magnitude of the effect. Values less than 0.035, between 0.035 and 0.07, and larger than 0.07 denote negligible, moderate, and large effect sizes, respectively (Jodoin and Gierl, 2001).

Item selection followed a two-step approach. First, the items exhibiting misfit or gender DIF were excluded. Second, four items were selected for each scale based on three criteria: the size of the factor loadings on the target dimension (and non-substantial factor loadings on non-target dimensions), the appropriateness of their location on the latent trait continuum (as indicated by the GRM threshold parameters), and the relevance of the item content to the dimension. Keeping four items per dimension is commonly recommended in the literature (Harvey et al., 1985; Hinkin, 1998) and ensures that the instrument is short.

The analyses described in this section were performed using the packages “psych” (Revelle, 2024), “mirt” (Chalmers et al., 2018), and “lordif” (Choi, 2016) for the open-source statistical environment R (R Core Team, 2018).

#### Validation of the MSSAT

The factor structure of the resulting scale was verified through a confirmatory factor analysis (CFA) conducted on a second subsample (*N* = 320; mean age = 37.53, *SD* = 13.83; males = 130, 40.6%). The model was run using Mplus7 (Muthén and Muthén, 2012) and the robust maximum likelihood estimator (MLR; Yuan and Bentler, 2000). Multiple-group CFAs were also performed on the entire dataset (*N* = 639) to test configural (same configuration of significant and nonsignificant factor loadings), metric (equality of factor loadings), and scalar (equality of both factor loadings and item intercepts) invariance across gender. To evaluate the goodness of fit of the CFAs models, several fit indexes were inspected: χ^2^, comparative fit index (CFI), standardized root mean square residual (SRMR), and root mean square error of approximation (RMSEA). A good fit is indicated by nonsignificant (*p* ≥ 0.05) χ^2^ values. Since this statistic is sensitive to sample size, the other fit measures were also inspected. CFI values close to 0.95 (0.90–0.95 for reasonable fit), and SRMR and RMSEA smaller than 0.06 (0.06–0.08 for reasonable fit) were considered indicative of adequate fit (Marsh et al., 2004). For testing the equivalence of nested models in gender invariance, the chi-square difference test (Δχ^2^) and the test of change in CFI (ΔCFI) were used. Invariance is indicated by a nonsignificant Δχ^2^ and by ΔCFIs lower than or equal to |0.01| (Cheung and Rensvold, 2002).

Reliability was verified through composite reliability (CR) coefficients. CR is conceptually similar to Cronbach’s *α* as it represents the ratio of true variance to total variance, but it is often considered a better index of internal consistency (Raykov, 2001). A CR value of at least 0.6 denotes satisfactory internal consistency (Bagozzi and Yi, 1988; Bentler, 2009).

Construct validity was verified by examining correlations between the seven soft skills of the MSSAT and three other constructs: job satisfaction, self-reported performance, and burnout. Based on the findings in the literature, initiative-resourcefulness, assertiveness, conflict management, communication skills, integrity, and adaptive decision making were expect to correlate positively with job satisfaction and self-reported performance, and negatively with burnout (e.g., Agba, 2018; Akla and Indradewa, 2022; Butt and Zahid, 2015; Byrnes, 2013; Ceschi et al., 2017; Ones et al., 1993; Keerativutisest and Hanson, 2017). The reverse pattern of correlations was expected for the maladaptive facets of decision-making, which were expected to correlate negatively with job satisfaction and self-reported performance, and positively with burnout (e.g., Allwood and Salo, 2012; Ceschi et al., 2017; Valieva, 2020).

To further explore the relationships between soft skills and the three considered work outcomes, network analysis was run. This approach involves estimating a network structure consisting of nodes and edges. The nodes represent the objects under analysis (i.e., scores on the considered scales), while the edges represent the relationships between them (i.e., regularized partial correlations between nodes, given all the other nodes in the network; Epskamp and Fried, 2018). Network analysis is a valuable approach, as it allows for easily exploring the interplay and interconnections among a large number of variables within a theoretical network. In this work, the network structure was built by including the scores on all the soft skills scales and the scores on the measures of burnout, job satisfaction, and self-reported performance. Based on the findings in the literature, soft skills are expected to promote better work outcomes (job satisfaction and better performance) while buffering the occurrence of burnout through direct associations and complex patterns of interconnections (Akla and Indradewa, 2022; Dehghan and Ma’toufi, 2016; Inwald et al., 1991; Tănase et al., 2012; Valieva, 2020). Therefore, network analysis is expected to provide additional contributions beyond simple correlational analyses by unveiling how soft skills interact in promoting positive work outcomes. This in turn would help to define the nomological network of soft skills (Bagozzi, 1981). In running network analysis, three common centrality indices were computed, namely betweenness, strength, and closeness. Strength centrality indicates the extent to which a node is connected with the other nodes within the network (the strength, in absolute value, of the direct connection of a node to the other nodes). High strength centrality indicates that a node is connected to many other network nodes. Closeness centrality evaluates how much a node is close to the other nodes in the network, including indirect connections. Nodes with large closeness centrality values are characterized by short paths linking them to the other nodes. Betweenness centrality captures the role of a node in connecting the other nodes within the network (Epskamp et al., 2018). Large values indicate that a node serves as a ‘bridge’ between the nodes in the network. These indices quantify the relevance of each variable in relation to the other variables within the network and provide additional relevant information beyond what is observed in other analyses. In this work, network analysis was run on the total sample (*N* = 639) using the EBICglasso (Extended Bayesian Information Criterion Graphical Least Absolute Shrinkage and Selection Operator; Foygel and Drton, 2010; Friedman et al., 2008) estimation method (tuning parameter was set to 0.5). Edges weights (i.e., partial correlation coefficients) were interpreted according to Ferguson’s (2016) guidelines, where values less than or equal to 0.2, from 0.2 to 0.5, and larger than 0.5 are considered as small, moderate, and large, respectively.

## Results

### Development of the MSSAT

Concerning the scale evaluating interpersonal skills, in line with expectations, the MAP test suggested to retain three factors. Consequently, an EFA and a multidimensional GRM with three factors were run. The results are reported in Table 1. All items showed substantial factor loadings on the intended dimension and non-substantial factor loadings on the other factors. No one item showed misfit, while six items showed uniform gender DIF (Init_16, Init_21, Init_36, Asse_32, Conf_15, and Conf_30; Init_16 exhibited nonuniform DIF as well). However, the effect size of DIF was negligible for all of them (Table 1). Since no item showed cross-loadings, misfit or noticeable DIF, four items were selected for each subscale by considering the magnitude of factor loadings, the item location on the latent trait continuum, and the item content. Following these criteria, items Init_11, Init_16, Init_21 and Init_36 were selected for the initiative-resourcefulness subscale; items Asse_2, Asse_12, Asse_22, and Asse_37 were selected for the assertiveness subscale; and items Conf_10, Conf_20, Conf_25, and Conf_35 were selected for the conflict management subscale.

**Table 1:** EFA factor loadings, GRM parameter estimates, fit indices, and gender DIF statistics for the 21 items of the three interpersonal skills subscales (calibration dataset, *N* = 319).

Item	Selected item	EFA			IRT						Fit			Gender DIF			
		λ1	λ2	λ3	a1	a2	a3	d1	d2	d3	S-χ^2^	*df*	*p*	U_DIF	NU_DIF	ES_U_DIF	ES_NU_DIF
Init_1		0.390	0.080	0.150	−0.375	0.364	−0.871	2.643	0.912	−1.116	60.514	58	0.385	0.367	0.365	0.003	0.001
Init_11	✓	0.700	0.300	0.100	−0.424	1.512	−2.093	5.415	2.459	−0.754	34.868	38	0.615	0.593	0.947	0.001	0.000
Init_16	✓	0.670	0.020	−0.060	0.109	0.600	−2.081	4.712	2.607	−0.982	46.945	44	0.353	**0.021**	**0.013**	0.011	0.009
Init_21	✓	0.650	0.190	0.020	−0.030	0.828	−1.604	2.610	0.077	−2.929	43.910	49	0.679	**0.001**	0.915	0.016	0.000
Init_32		0.520	−0.070	−0.070	0.128	0.200	−1.461	4.176	2.498	−0.668	46.284	45	0.419	0.345	0.147	0.003	0.003
Init_36	✓	0.410	0.220	0.230	−0.563	0.722	−0.818	2.666	0.803	−1.027	41.110	49	0.781	**0.003**	0.134	0.014	0.003
Asse_2	✓	−0.010	0.660	−0.080	0.364	1.842	0.370	3.815	0.980	−2.155	39.361	45	0.709	0.809	0.941	0.001	0.000
Asse_7		0.020	0.630	0.110	−0.284	1.607	0.240	3.986	1.329	−0.607	49.985	40	0.134	0.414	0.219	0.002	0.002
Asse_12	✓	−0.060	0.590	0.100	−0.311	1.654	0.445	4.904	2.733	0.439	39.231	37	0.370	0.851	0.964	0.001	0.000
Asse_17		0.150	0.630	0.010	−0.039	1.995	−0.124	7.469	3.084	0.109	47.123	34	0.067	0.421	0.239	0.003	0.002
Asse_22	✓	−0.020	0.680	0.110	−0.214	2.004	0.498	4.721	1.843	−1.710	47.343	39	0.169	0.401	0.243	0.003	0.002
Asse_27		0.060	0.640	0.060	−0.051	1.969	0.262	4.862	2.538	−0.976	43.853	37	0.204	0.812	0.519	0.001	0.001
Asse_32		0.020	0.720	0.100	−0.252	2.450	0.473	4.544	1.371	−1.602	28.463	43	0.957	**0.001**	0.654	0.017	0.000
Asse_37	✓	0.100	0.800	−0.040	0.293	3.529	0.335	6.748	3.230	−1.158	31.606	35	0.633	0.627	0.405	0.001	0.001
Conf_10	✓	−0.030	0.100	0.490	−1.174	0.296	0.184	2.251	0.153	−2.043	52.170	51	0.428	0.858	0.613	0.000	0.000
Conf_15		0.100	0.230	0.490	−1.234	0.687	−0.077	3.817	1.296	−1.382	44.014	41	0.345	**0.048**	0.140	0.008	0.003
Conf_20	✓	0.150	0.090	0.590	−1.599	0.427	−0.330	5.234	1.627	−1.778	32.956	41	0.810	0.388	0.744	0.003	0.000
Conf_25	✓	−0.020	0.030	0.600	−1.498	0.143	0.096	3.444	0.924	−1.379	49.354	46	0.341	0.841	0.557	0.000	0.000
Conf_30		−0.020	0.150	0.600	−1.571	0.408	0.182	3.767	1.082	−1.662	46.930	43	0.315	**0.004**	0.070	0.015	0.004
Conf_35	✓	0.060	0.230	0.620	−1.826	0.740	0.000	5.125	2.476	−0.791	51.739	38	0.068	0.723	0.514	0.001	0.001
Conf_40		0.010	−0.050	0.590	−1.577	0.000	0.000	3.290	1.196	−1.167	56.420	54	0.385	0.345	0.586	0.003	0.000

λ1, λ2, λ3 = EFA factor loadings; a1, a2, a3 = GRM discrimination parameters; d1, d2, d3 = GRM threshold parameters. S-χ^2^ = Signed chi square (item fit index); *df* = degrees of freedom of S-χ^2^; *p* = *p* value of S-χ^2^; NU_DIF = nonuniform gender bias (significant DIF in bold); U_DIF = uniform gender bias (significant DIF in bold); ES_U_DIF = effect size of uniform gender DIF; ES_NU_DIF = effect size of nonuniform gender DIF.

Concerning the scale evaluating communication skills, in line with expectations, the MAP test suggested to retain one factor. Therefore, a single factor EFA and a unidimensional GRM were run. The results are reported in Table 2. All items reported substantial factor loadings on the intended dimension and no item showed uniform or nonuniform gender DIF. However, item Comm_2 exhibited misfit. After having excluded this item from the pool, the four items required to compose the interpersonal communication skill scale were selected considering the magnitude of their factor loadings, their location on the latent trait continuum, and their content. Following these criteria, items Comm_1, Comm_8, Comm_9, and Comm_10 were selected.

**Table 2:** EFA factor loadings, GRM parameter estimates, fit indices, and gender DIF statistics for the nine items of the interpersonal communication skills scale (calibration dataset, *N* = 319).

Item	Selected item	EFA	IRT				Fit			Gender DIF			
		λ1	a1	d1	d2	d3	S-χ^2^	*df*	*p*	U_DIF	NU_DIF	ES_U_DIF	ES_NU_DIF
Comm_1	✓	0.560	1.422	3.486	1.261	−1.754	33.083	24	0.102	0.563	0.823	0.002	0.000
Comm_2		0.300	0.672	1.738	0.280	−1.648	53.154	33	**0.015**	0.465	0.586	0.002	0.000
Comm_3		0.680	2.086	4.315	1.704	−1.870	30.439	21	0.084	0.410	0.638	0.002	0.000
Comm_7		0.380	0.880	2.820	0.914	−1.756	41.088	29	0.068	0.061	0.082	0.007	0.004
Comm_8	✓	0.500	1.256	5.304	2.463	−0.962	26.364	19	0.120	0.162	0.063	0.006	0.006
Comm_9	✓	0.740	2.767	5.286	2.388	−1.858	13.239	20	0.867	0.432	0.611	0.002	0.000
Comm_10	✓	0.700	2.226	5.908	2.197	−2.374	15.359	15	0.426	0.123	0.431	0.007	0.001
Comm_14		0.600	1.784	4.857	3.073	−0.376	12.430	19	0.866	0.548	0.525	0.002	0.001
Comm_16		0.660	2.091	4.684	2.163	−1.561	23.678	21	0.309	0.224	0.085	0.004	0.004

λ1 = EFA factor loadings; a1 = GRM discrimination parameters; d1, d2, d3 = GRM threshold parameters; S-χ^2^ = Signed chi square (item fit index); *df* = degrees of freedom of S-χ^2^; *p* = *p* value of S-χ^2^ (significant misfit in bold); NU_DIF = nonuniform gender bias (significant DIF in bold); U_DIF = uniform gender bias (significant DIF in bold); ES_U_DIF = effect size of uniform gender DIF; ES_NU_DIF = effect size of nonuniform gender DIF.

With regard to the decision-making scale, the MAP test suggested to retain two factors. This result was unexpected because the items were anticipated to tap the four dimensions of vigilance, hypervigilance, buck-passing, and procrastination. However, following MAP indications, EFA and exploratory multidimensional GRM with two factors were run. The results demonstrated that all the items related to vigilance loaded onto a single common factor, while the remaining items, which were related to hypervigilance, buck-passing, and procrastination, loaded onto the second factor. In other words, the results showed a structure where the adaptive style of decision-making loaded on one factor and the three maladaptive styles were grouped on a single common dimension. In the maladaptive decision-making factor, two items (items MDM_7, and MDM_20 tapping, respectively, the buck-passing and hypervigilance facets of maladaptive decision-making) showed misfit and three items exhibited uniform gender DIF (MDM_12, MDM_17, and MDM_22, which tapped the buck-passing, procrastination, and hypervigilance facets of maladaptive decision-making, respectively) of negligible size (Table 3). In the adaptive decision-making factor, no item showed misfit or DIF. Since only two items from the maladaptive decision-making style were excluded from the selection due to misfit, the eight items to include in the two final decision-making subscales were selected based on the magnitude of their factor loadings, their location on the latent trait continuum, and their content. The items selected for the adaptive subscale were ADM_1, ADM_3, ADM_4, and ADM_6, while those selected for the maladaptive subscale were MDM_11, MDM_13, MDM_15, and MDM_21 (MDM_13 and MDM_15 pertain to procrastination, MDM_11 to buck-passing, and MDM_21 to hypervigilance).

**Table 3:** EFA factor loadings, GRM parameter estimates, fit indices, and gender DIF statistics for the 22 items of the two decision-making subscales (calibration dataset, *N* = 319).

Item	Selected item	EFA		IRT					Fit			Gender DIF			
		λ1	λ2	a1	a2	d1	d2	d3	S-χ^2^	*df*	*p*	U_DIF	NU_DIF	ES_U_DIF	ES_NU_DIF
ADM_1	✓	−0.110	0.710	−0.897	2.199	6.655	4.037	0.342	38.013	42	0.647	0.849	0.567	0.001	0.001
ADM_2		0.020	0.670	−1.163	1.802	6.236	3.601	0.390	39.391	44	0.669	0.418	0.919	0.003	0.000
ADM_3	✓	0.140	0.620	−1.421	1.480	6.318	3.670	0.451	45.438	39	0.222	0.388	0.476	0.003	0.001
ADM_4	✓	−0.240	0.590	−0.295	2.075	6.233	4.193	0.714	44.226	34	0.113	0.975	0.940	0.000	0.000
ADM_5		−0.180	0.510	−0.335	1.627	5.323	3.132	−0.054	49.307	45	0.305	0.894	0.643	0.000	0.000
ADM_6	✓	−0.120	0.670	−0.819	2.154	8.159	3.945	0.936	53.449	41	0.092	0.501	0.288	0.003	0.002
MDM_7		0.610	−0.210	−1.192	−1.305	0.608	−1.823	−4.141	66.073	45	**0.022**	0.571	0.949	0.002	0.000
MDM_8		0.720	−0.140	−2.015	−1.782	0.104	−2.829	−5.503	31.722	37	0.715	0.367	0.237	0.003	0.002
MDM_9		0.720	−0.090	−1.972	−1.467	0.445	−2.404	−4.840	47.270	40	0.200	0.839	0.555	0.001	0.001
MDM_10		0.620	−0.080	−1.264	−0.936	1.336	−0.880	−3.332	49.568	47	0.371	0.530	0.316	0.002	0.001
MDM_11	✓	0.610	0.160	−1.558	−0.392	2.589	0.165	−2.256	51.092	48	0.353	0.246	0.998	0.003	0.000
MDM_12		0.400	0.190	−0.916	0.000	2.352	0.200	−1.378	63.068	60	0.368	**0.002**	0.436	0.015	0.001
MDM_13	✓	0.660	−0.080	−1.461	−1.105	1.339	−1.227	−3.889	42.230	44	0.548	0.253	0.113	0.004	0.003
MDM_14		0.710	0.010	−1.865	−0.944	2.275	−0.289	−2.592	60.187	48	0.112	0.509	0.330	0.002	0.001
MDM_15	✓	0.540	0.170	−1.314	−0.247	2.078	−0.037	−2.307	55.678	56	0.487	0.995	0.949	0.000	0.000
MDM_16		0.590	0.000	−1.346	−0.723	1.180	−0.868	−3.148	49.549	45	0.297	0.167	0.166	0.004	0.002
MDM_17		0.440	0.220	−1.065	0.008	2.741	0.849	−1.447	62.447	56	0.258	**0.001**	0.942	0.018	0.000
MDM_18		0.540	−0.120	−1.057	−0.978	0.676	−1.375	−3.007	59.403	48	0.125	0.917	0.958	0.000	0.000
MDM_19		0.400	0.050	−0.864	−0.350	1.439	−0.176	−2.205	53.499	42	0.110	0.474	0.614	0.002	0.000
MDM_20		0.700	0.040	−1.934	−1.053	0.548	−1.588	−4.024	64.883	43	**0.017**	0.172	0.202	0.004	0.002
MDM_21	✓	0.770	−0.050	−2.375	−1.534	0.738	−2.474	−5.427	34.792	36	0.526	0.781	0.521	0.001	0.001
MDM_22		0.650	0.060	−1.599	−0.737	1.947	−0.302	−2.834	50.687	52	0.526	**0.024**	0.088	0.009	0.004

λ1, λ2 = EFA factor loadings; a1, a2 = GRM discrimination parameters; d1, d2, d3 = GRM threshold parameters. S-χ^2^ = Signed chi square (item fit index); *df* = degrees of freedom of S-χ^2^; *p* = *p* value of S-χ^2^ (significant misfit in bold); NU_DIF = nonuniform gender bias (significant DIF in bold); U_DIF = uniform gender bias (significant DIF in bold); ES_U_DIF = effect size of uniform gender DIF; ES_NU_DIF = effect size of nonuniform gender DIF; ADM = adaptive decision making; MDM = maladaptive decision making.

Finally, for the integrity scale, in line with expectations, the MAP test suggested a unidimensional structure. Therefore, a single factor EFA and a unidimensional GRM were run. The results are reported in Table 4. All items reported substantial loadings on the latent factor. Item Inte_6 exhibited misfit while items Inte_2 (uniform) and Inte_18 (uniform and nonuniform) showed gender DIF of negligible size. After having discarded Inte_2 due to misfit, the four items of the short integrity scale were selected considering the magnitude of their factor loadings, their location on the latent trait continuum, and the item content. Following these criteria, items Inte_1, Inte_3, Inte_12, and Inte_17 were selected.

**Table 4:** EFA factor loadings, GRM parameter estimates, fit indices, and gender DIF statistics for the 12 items of the integrity scale (calibration dataset, *N* = 319).

Item	Selected item	EFA	IRT				Fit			Gender DIF			
		λ1	a1	d1	d2	d3	S-χ^2^	*df*	*p*	U_DIF	NU_DIF	ES_U_DIF	ES_NU_DIF
Inte_1	✓	0.610	1.783	4.570	2.802	0.384	23.594	25	0.543	0.606	0.817	0.002	0.000
Inte_2		0.330	0.670	2.758	0.901	−0.983	38.628	43	0.661	**0.022**	0.160	0.010	0.003
Inte_3	✓	0.550	1.372	3.180	1.043	−0.905	47.049	37	0.125	0.841	0.658	0.000	0.000
Inte_4		0.260	0.546	3.022	1.277	−0.730	44.798	37	0.177	0.100	0.852	0.007	0.000
Inte_6		0.530	1.255	4.853	1.868	−0.439	51.615	28	**0.004**	0.747	0.531	0.001	0.001
Inte_12	✓	0.580	1.492	3.300	1.230	−0.335	28.006	36	0.827	0.707	0.844	0.001	0.000
Inte_13		0.650	2.084	5.031	3.145	0.140	18.718	23	0.717	0.506	0.451	0.002	0.001
Inte_14		0.540	1.474	4.821	2.573	0.419	34.432	26	0.124	0.615	0.486	0.002	0.001
Inte_15		0.580	1.418	2.726	0.840	−1.056	39.182	40	0.507	0.068	0.562	0.006	0.000
Inte_16		0.250	0.523	2.834	1.318	−0.504	37.490	43	0.708	0.891	0.687	0.000	0.000
Inte_17	✓	0.660	2.341	5.529	3.707	0.500	23.432	22	0.378	0.178	0.135	0.006	0.004
Inte_18		0.250	0.630	3.202	1.796	−0.191	41.541	36	0.242	**0.003**	**0.001**	0.016	0.015

λ1 = EFA factor loadings; a1 = GRM discrimination parameters; d1, d2, d3 = GRM threshold parameters. S-χ^2^ = Signed chi square (item fit index); *df* = degrees of freedom of S-χ^2^; *p* = *p* value of S-χ^2^ (significant misfit in bold); NU_DIF = nonuniform gender bias (significant DIF in bold); U_DIF = uniform gender bias (significant DIF in bold); ES_U_DIF = effect size of uniform gender DIF; ES_NU_DIF = effect size of nonuniform gender DIF.

### Validation of the MSSAT

The factor structure of the scale built on the first subsample was tested through CFA on the second subsample (*N* = 320; the items of the MSSAT are available in the Appendix). In particular, a 7-factor structure with four indicators for each dimension was specified. The model showed satisfactory fit indices: χ^2^(329) = 476.107, *p* < 0.001; CFI = 0.927; RMSEA = 0.037 [0.030, 0.045]; SRMR = 0.059. All items loaded with large coefficients on the intended factor and factor intercorrelations were moderate in size (Table 5). Tested on the entire dataset (*N* = 639), measurement invariance (configural, metric, and scalar) across gender was also supported (Table 6). Internal consistency was satisfactory for all scales (*CR*s from 0.69 to 0.78, see Table 5; Cronbach’s *α* from 0.67 to 0.76, see Table 7).

**Table 5:** CFA factor loadings, factor correlations, and composite reliability coefficients (validation dataset, *N* = 320).

	INIT	ASSE	CONF	COMM	ADM	MDM	INTE
INIT_11	0.688						
INIT_16	0.365						
INIT_21	0.695						
INIT_36	0.755						
ASSE_2		0.715					
ASSE_12		0.513					
ASSE_22		0.698					
ASSE_37		0.747					
CONF_10			0.456				
CONF_20			0.715				
CONF_25			0.576				
CONF_35			0.622				
COMM_1				0.742			
COMM_8				0.637			
COMM_9				0.668			
COMM_10				0.705			
ADM_1					0.703		
ADM_3					0.669		
ADM_4					0.651		
ADM_6					0.678		
MDM_11						0.629	
MDM_17						0.772	
MDM_20						0.642	
MDM_22						0.640	
INTE_1							0.649
INTE_3							0.581
INTE_12							0.765
INTE_17							0.383
Factor correlations							
ASSE	0.381						
CONF	0.252	0.29					
COMM	0.446	0.464	488				
ADM	0.204**	0.232**	0.386	0.349			
MDM	−0.36	−0.448	−0.237	−0.417	−0.112†		
INTE	−0.094†	0.064†	0.232**	0.237**	0.248**	−0.303	
*CR*	0.73	0.77	0.69	0.78	0.77	0.77	0.69

The table presents the factor loadings and the factor correlations of the CFA model concerning the MSSAT. INIT, initiative-resourcefulness; ASSE, assertiveness; CONF, conflict management; COMM, interpersonal communication skills; ADM, adaptive decision making; MDM, maladaptive decision making; INTE, integrity. All coefficients are significant at *p* ≤ 0.001, excluding those marked with **(*p* ≤ 0.01) and †(*p* > 0.05).

**Table 6:** Fit indices of multiple-groups factor analyses run to test the gender invariance of the MSSAT (entire dataset; *N* = 639).

Model	χ^2^	*df*	*p*	RMSEA	CFI	SRMR	∆CFI	∆χ^2^	*df*	*p*
Configural	976.651	658	0	0.039	0.917	0.061				
Metric	994.669	679	0	0.038	0.917	0.064	0.000	19.196	21	0.5725
Scalar	1,024.397	700	0	0.038	0.915	0.064	0.002	29.618	21	0.0999

RMSEA, root mean square error of approximation; CFI, comparative fit index; SRMR, standardized root mean square residual; Δχ^2^, chi square difference test; ΔCFI, test of change in CFI.

**Table 7:** Descriptive statistics, reliability, correlations between all variables (entire dataset, *N* = 639).

Variable	Mean	SD	*α*	1	2	3	4	5	6	7	8	9	10
1. Initiative-resourcefulness	2.922	0.649	0.72	—									
2. Assertiveness	3.047	0.620	0.75	0.258	—								
3. Conflict management	2.887	0.580	0.67	0.177	0.182	—							
4. Communication	2.991	0.574	0.75	0.342	0.308	0.304	—						
5. Adaptive decision making	3.467	0.511	0.76	0.183	0.169	0.228	0.245	—					
6. Maladaptive decision making	2.215	0.679	0.74	−0.216	−0.376	−0.176	−0.249	−0.052	—				
7. Integrity	3.202	0.622	0.70	−0.060	0.080	0.252	0.121	0.196	−0.234	—			
8. Performance	8.031	1.129	0.86	0.142	0.203	0.143	0.290	0.193	−0.221	0.190	—		
9. Burnout	1.533	0.489	0.84	−0.151	−0.213	−0.106	−0.219	−0.206	0.343	−0.183	−0.388	—	
10. Job satisfaction	2.883	0.676	0.82	0.122	0.130	0.146	0.253	0.145	−0.250	0.215	0.343	−0.625	—

*α = Cronbach’s alpha coefficients.*

All correlations between the seven soft kills, job satisfaction, self-reported performance, and burnout were consistent with expectations (Table 7). In particular, the soft skills were positively associated with job satisfaction and performance, and negatively associated with burnout. The only exception was the maladaptive facet of decision-making, which showed an inverted pattern of relationships, as expected. Although these correlations were weak in strength, they were statistically significant and in line with expectations. This result supports the construct validity of the seven soft skills subscales.

**Figure 1:**
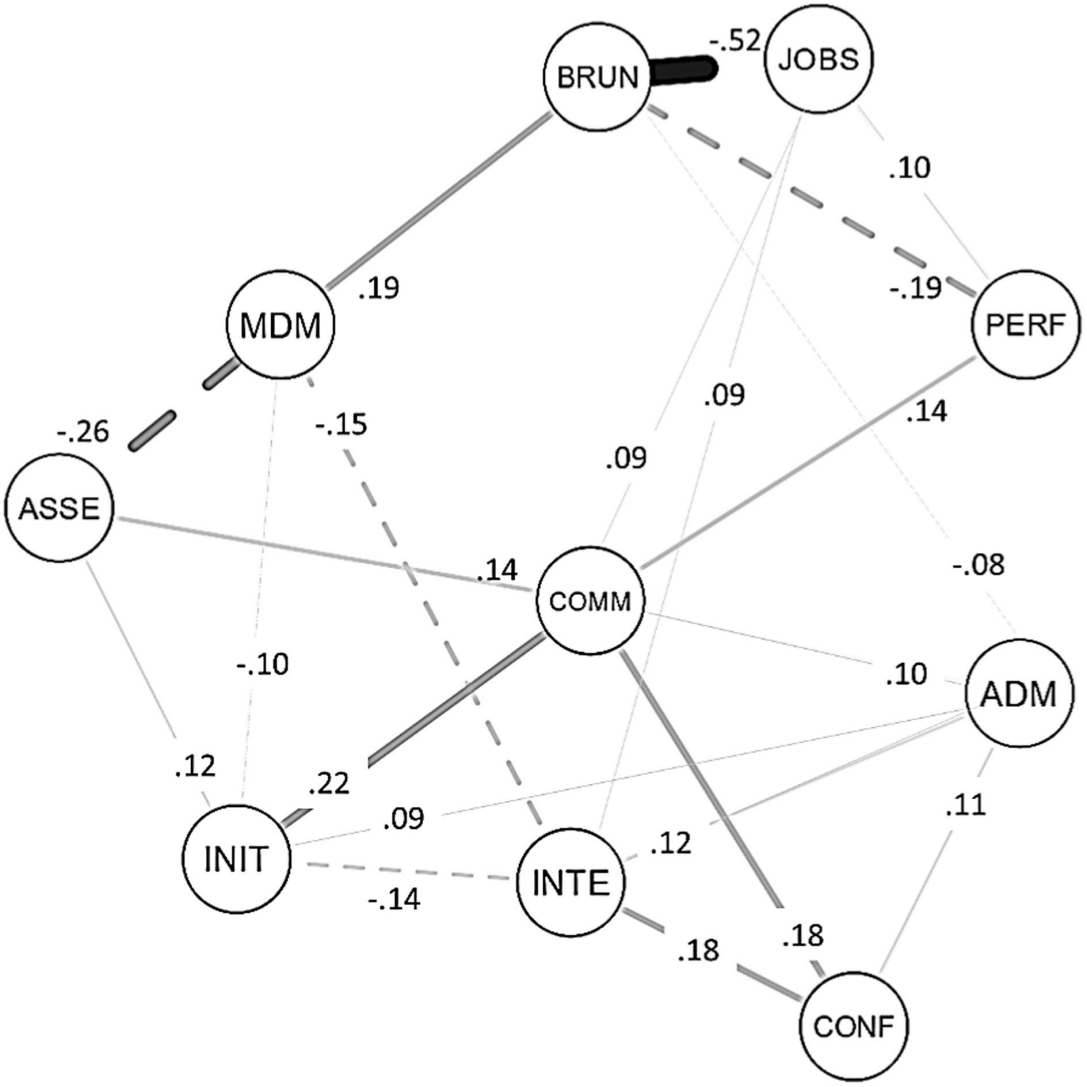
Network structure (entire dataset*, N* = 639). Network originating from the seven soft skills measured using the MSSAT, plus burnout, job satisfaction, and performance. Solid lines indicate positive connections while dotted lines indicates negative connections. Thicker lines represent stronger connections while thinner represent weaker connections. For the sake of simplicity, only significant (*p* < 0.05) coefficients ≥ |0.08| were reported in the figure. INIT, Initiative-resourcefulness; ASSE, assertiveness; CONF, conflict management; COMM, interpersonal communication skills; ADM, adaptive decision making; MDM, maladaptive decision making; INTE, integrity; PERF, performance; BURN, burnout; JOBS, job satisfaction.

The network structure deriving from the 10 variables entered in the model (the seven soft skills measured with the MSSAT, plus burnout, job satisfaction, and performance) is represented in Figure 1. The structure includes 10 nodes (i.e., one for each variable) and 36/45 non-zero edges (sparsity of 0.200). Overall, the analysis showed edges of small to moderate size (average weights = |0.09|; see Table 8). The examination of the network structure revealed that soft skills are all interrelated with each other and associated with burnout, job satisfaction, and performance, in the expected directions. Moreover, the analysis revealed that soft skills impact work outcomes not only through direct associations but also through their interplay. For instance, with regard to performance, a significant positive edge was observed only with communication skills. However, communication skills are also associated with interpersonal skills (initiative-resourcefulness, assertiveness, conflict management), and with the adaptive facet of decision making. This pattern of relationships among the variables suggests that the positive associations of adaptive decision-making and interpersonal skills with performance that emerged in the correlational analyses may be attributed to the role of interpersonal communication skills, which may serve as a bridge connecting them. Analogously, only two soft skills are directly associated with job satisfaction, namely communication skills and integrity. However, these two variables are linked to many other skills within the network structure, enabling them to connect different skills to job satisfaction. Communication skills are particularly important in linking job satisfaction with interpersonal skills (i.e., initiative-resourcefulness, assertiveness, and conflict management) and the adaptive facet of decision making. In contrast, integrity is relevant in linking job satisfaction with decision-making (both adaptive and maladaptive), initiative-resourcefulness, and conflict management. Finally, regarding burnout, only two direct associations were observed with the two facets of decision making. Specifically, as predicted, the maladaptive facet showed a positive association, while the adaptive facet showed a negative association. The examination of the network structure reveals that adaptive and maladaptive decision making are also linked to other soft skills, serving as a bridge between them and burnout. In particular, the adaptive facet of decision-making negatively links burnout with communication, initiative-resourcefulness, conflict management and integrity, while the maladaptive facet of the construct positively links burnout with assertiveness and integrity.

**Table 8:** Weight matrix and centrality measures from network analysis (entire dataset, *N* = 639).

Variable	Betweenness	Closeness	Strength	1	2	3	4	5	6	7	8	9	10
1. Initiative-resourcefulness	0.000	0.847	0.739	—									
2. Assertiveness	0.143	0.853	0.680	**0.115**	—								
3. Conflict management	0.143	0.789	0.609	0.063	0.040	—							
4. Communication	1.000	1.000	0.919	**0.222**	**0.141**	**0.182**	—						
5. Adaptive decision making	0.000	0.719	0.691	**0.092**	0.057	**0.114**	0.100	—					
6. Maladaptive decision making	0.857	0.998	0.890	**−0.100**	**−0.265**	−0.034	−0.056	0.074	—				
7. Integrity	0.429	0.929	0.740	**−0.135**	0.000	0.178	0.000	0.120	−0.154	—			
8. Performance	0.143	0.809	0.643	0.007	0.052	0.000	**0.144**	0.060	−0.028	0.073	—		
9. Burnout	0.714	0.883	1.000	−0.015	−0.020	0.000	0.000	**−0.084**	**0.189**	0.000	**−0.186**	—	
10. Job satisfaction	0.000	0.826	0.797	0.000	0.000	0.006	**0.087**	0.000	−0.003	**0.090**	**0.102**	**−0.520**	—

The values in the table are partial correlation coefficients. Significant (*p* < 0.05) partial correlation coefficients ≥ |The values in the table are partial correlation coefficients. Significant (p < 0.05) partial correlation coefficients ≥ |0.08| are in bold.|The values in the table are partial correlation coefficients. Significant (p < 0.05) partial correlation coefficients ≥ |0.08| are in bold.

The inspection of centrality indices suggests that interpersonal communication skills, maladaptive decision-making, and integrity are crucial skills for workers’ performance and well-being. These variables, in fact, showed the largest values of betweenness, closeness, and strength centrality (Table 8), indicating that they had the strongest paths with the other variables within the network and a crucial role in connecting them.

## Discussion

In this work, a scale for assessing soft skills in organizational contexts was developed and validated following best practices in the literature (Boateng et al., 2018; Hinkin, 1998). An initial pool of 64 items was created by selecting and adapting items from existing instruments (Buhrmester et al., 1988; Hecht, 1978; Mann et al., 1997; Schlenker, 2008). The initial item pool was analyzed using detailed item-level methods on data from a first sample of individuals. These analyses allowed for identifying the four best items for each dimension to be included in the MSSAT.

The instrument consists of 28 items (see “Appendix”) that assess seven soft skills—initiative-resourcefulness, assertiveness, conflict management, adaptive decision-making, maladaptive decision-making, communication, and integrity—organized into four main domains (interpersonal relations, communication, decision-making, and integrity). The factor structure of the scale was confirmed on a second independent sample. All subscales showed satisfactory reliability and full scalar invariance across gender was supported. This last property is particularly valuable, as it allows for confidently using the scale to assess both men and women, and to make meaningful comparisons between them (Anselmi et al., 2022; Colledani, 2018; Fagnani et al., 2021; Vandenberg and Lance, 2000). The validity of the scale was verified by examining the correlations between the scores on the seven soft skills and measures of burnout, performance, and job satisfaction. The results showed correlations that were consistent with expectations (e.g., Dehghan and Ma’toufi, 2016; Keerativutisest and Hanson, 2017; Posthuma and Maertz, 2003; Valieva, 2020), supporting the construct validity of the MSSAT. Regarding validity, a further contribution of this work is the examination of the nomological network of soft skills with respect to the three work-related outcomes considered, which was carried out using network analysis. Network analysis represents a novel yet effective approach for exploring the nomological network of a large set of variables, as it allows for the simultaneous investigation of the complex network of interactions that connect variables. Overall, the analysis confirmed the positive role of soft skills in improving employee performance and well-being. Moreover, it revealed that soft skills are not only directly related to work outcomes, but also through complex patterns of relationships. Communication skills, maladaptive decision-making, and integrity were identified as pivotal resources based on the analysis of centrality indices. These skills are directly related to positive work outcomes and also serve to connect many other soft skills in the network. In particular, the results emphasize the critical role of communication skills and suggest that they can be viewed as key competencies capable of supporting the development of other soft skills, which ultimately contribute to professional flourishing.

Another interesting finding of the present work pertains to the substantial influence of integrity in work contexts. Indeed, this variable has long been recognized as beneficial and relevant in organizational contexts (Inwald et al., 1991; Luther, 2000; Ones et al., 1993; Posthuma and Maertz, 2003), but its role has not been deeply investigated. The results of the present work show that integrity is strongly related to many other soft skills and thus may play an important role in determining employee satisfaction.

Although the results of the present work are interesting, further investigation is needed. Future studies should focus on confirming the nomological network that emerged in this analysis and should also attempt to confirm our findings in different occupational or cultural contexts.

One of the strengths of the developed scale is its deliberately general wording. This feature makes the scale applicable in various work contexts and could potentially expand its usefulness to non-work settings, such as schools. However, further research is needed to determine the suitability of the instrument in such contexts. For example, future research could test the invariance of the instrument across different job positions and settings (e.g., organizational versus school).

Future studies would be devoted to developing a shorter version of the MSSAT, as well as another version that assesses additional key soft skills. Professionals in different organizational contexts could administer only those subscales that assess the soft skills most relevant to their area of interest.

Although further studies are needed to confirm the validity of the MSSAT, the scale appears to be a promising tool for assessing soft skills in organizational settings. It should be noted that the main limitation of the present work is its exclusive reliance on self-reported data, which are susceptible to biases such as social desirability. Future research should test the validity of the scale by considering other, more objective measures, such as peer, supervisor and manager ratings on outcome measures (e.g., work performance, absenteeism), or implicit measures (e.g., Carton and Hofer, 2006; Colledani and Camperio Ciani, 2021; Harris and Schaubroeck, 1988). In addition, the cross-sectional nature of this study limits the understanding of the predictive validity of the MSSAT. Longitudinal research is essential to examine the scale ability to predict long-term outcomes such as career advancement, and would support the usefulness of the instrument as an effective tool for personnel selection and training design.

Despite the limitations mentioned above, the MSSAT emerges as a valuable tool for organizations due to its ability to quickly assess numerous soft skills with sufficient validity and reliability. These skills have been shown to be relevant in promoting better job satisfaction and work-related outcomes. The development of the MSSAT has significant implications for organizational practice. Assessing and developing soft skills is essential for personnel selection, career advancement, employability, and positive work-related outcomes (e.g., Agba, 2018; Akla and Indradewa, 2022; Hogan et al., 2013; Nusrat and Sultana, 2019; Poláková et al., 2023). By identifying areas for improvement, the MSSAT may help organizations to tailor training programs that enhance employees’ soft skills and consequently their professional flourishing.

## Data Availability

The datasets analyzed for this study can be found in the OSF repository: https://osf.io/6rqcm/?view_only=f9fcdef687454451b0593c6661bcf1a3.

## Appendix

Below you will read a series of statements describing common ways of behaving and thinking.

For each statement, please indicate the extent to which it reflects the way you usually behave and think.

Please, indicate your level of agreement with each statement, remembering that there are no right or wrong answers or tricks, only answers that do or do not correspond to your way of being and doing.

Answer quickly, without taking too long to think about the possible meanings of the statements.

Please, remember to answer all questions.

When answering the questions, please refer to the following scale:

1 = Strongly disagree

2 = Moderately disagree

3 = Moderately agree

4 = Strongly agree

**Table:**

INITIATIVE-RESOURCEFULNESS	
INIT_11	I have no problem starting a conversation with someone I do not know if I think they might turn out to be interesting.
INIT_16	I try to present myself as an interesting and pleasant person when I meet someone for the first time.
INIT_21	I have no difficulty in taking part in meetings or gatherings in which I do not know the people well and with whom I may have to interact.
INIT_36	I make the first move by introducing myself when I meet someone who might be interesting to meet.
ASSERTIVENESS	
ASSE_2	If I’m treated in a way I do not like, I speak up.
ASSE_12	I know how to say no to something I think is unreasonable.
ASSE_22	If someone does something that makes me feel uncomfortable, I point it out.
ASSE_37	I express my feelings when someone does something that upsets me.
CONFLICT MANAGEMENT	
CONF_10	When arguing with someone, I can let go of my resentment.
CONF_20	When arguing, I can put myself in the other person’s shoes and really try to understand how they feel.
CONF_25	I know how to avoid saying something which might lead to disagreeing and getting into a serious argument.
CONF_35	If I am angry with someone, I can accept that they may have a valid point of view, even if it is different from mine.
INTERPERSONAL COMMUNICATION	
COMM_1	In my recent conversations, the other person let me know that I was communicating effectively.
COMM_8	In my conversations, people usually show me that they have understood what I said.
COMM_9	I have been very happy with the conversations I have had recently.
COMM_10	In my recent conversations, the other person expressed a lot of interest in what I had to say.
ADAPTIVE DECISION MAKING	
ADM_1	I like to consider all of the alternatives when making decisions.
ADM_3	I consider how best to carry out a decision.
ADM_4	When making decisions, I like to collect a lot of information.
ADM_6	I take a lot of care before choosing.
MALADAPTIVE DECISION MAKING	
MDM_11	If a decision can be made by me or another person, I let the other person make it.
MDM_17	I put off making decisions.
MDM_20	The possibility that some small thing might go wrong causes me to swing abruptly in my preference.
MDM_22	After a decision is made, I spend a lot of time convincing myself it was correct.
INTEGRITY	
INTE_1	It is foolish to tell the truth when big profits can be made by lying. (R)
INTE_3	Regardless of concerns about principles, in today’s world you have to be practical, adapt to opportunities, and do what is most advantageous for you. (R)
INTE_12	Lying is sometimes necessary to accomplish important, worthwhile goals. (R)
INTE_17	One’s principles should not be compromised regardless of the possible gain.

The Italian version of the scale is available upon request from the corresponding author.

## References

[ref1] Abelha M. Fernandes S. Mesquita D. Seabra F. Ferreira-Oliveira A. T. (2020). Graduate employability and competence development in higher education—a systematic literature review using PRISMA. Sustain. For. 12:5900. doi: 10.3390/su12155900, PMID: 36706730

[ref2] Adhvaryu A. Kala N. Nyshadham A. (2023). Returns to on-the-job soft skills training. J. Polit. Econ. 131, 2165–2208. doi: 10.1086/724320

[ref3] Agba M. S. (2018). Interpersonal relationships and organizational performance: the Nigerian public sector in perspective. Indian J. Comm. Manag. Stud. IX, 75–86. doi: 10.18843/ijcms/v9i3/08

[ref4] Akla S. Indradewa R. (2022). The effect of soft skill, motivation and job satisfaction on employee performance through organizational commitment. BIRCI J. 5, 6070–6083. doi: 10.33258/birci.v5i1.432

[ref5] Allwood C. M. Salo I. (2012). Decision-making styles and stress. Int. J. Stress. Manag. 19, 34–47. doi: 10.1037/a0027420, PMID: 39357399

[ref6] Alsabbah M. Y. Ibrahim H. I. (2013). Employee competence (soft and hard) outcome of recruitment and selection process. Am. J. Econ. 3, 67–73. doi: 10.5923/c.economics.201301.12

[ref7] Anselmi P. Colledani D. Andreotti A. Robusto E. Fabbris L. Vian P.. (2022). An item response theory-based scoring of the south oaks gambling screen–revised adolescents. Assessment 29, 1381–1391. doi: 10.1177/10731911211017657, PMID: 34036842

[ref8] Asefer A. Abidin Z. (2021). Soft skills and graduates’ employability in the 21st century from employers’ perspectives: a review of literature. Int. J. Infrastruct. Res. Manag. 9, 44–59.

[ref9] Awan A. G. Saeed S. (2015). Conflict management and organizational performance: a case study of Askari Bank Ltd. Res. J. Finance Account. 6, 88–102.

[ref10] Bagozzi R. P. (1981). An examination of the validity of two models of attitude. Multivar. Behav. Res. 16, 323–359. doi: 10.1207/s15327906mbr1603_4 26815596

[ref11] Bagozzi R. P. Yi Y. (1988). On the evaluation of structural equation models. J. Acad. Mark. Sci. 16, 74–94. doi: 10.1007/BF02723327, PMID: 39395122

[ref12] Balcar J. (2016). Is it better to invest in hard or soft skills? Econ. Labour Relat. Rev. 27, 453–470. doi: 10.1177/1035304616674613

[ref9001] Bambacas M. Patrickson M. (2008). Interpersonal communication skills that enhance organisational commitment. J. Commun. Manag. 12, 51–72. doi: 10.1108/13632540810854235

[ref13] Bentler P. M. (2009). Alpha, dimension-free, and model-based internal consistency reliability. Psychometrika 74, 137–143. doi: 10.1007/s11336-008-9100-1, PMID: 20161430 PMC2786226

[ref14] Boateng G. O. Neilands T. B. Frongillo E. A. Melgar-Quiñonez H. R. Young S. L. (2018). Best practices for developing and validating scales for health, social, and behavioral research: a primer. Front. Public Health 6:149. doi: 10.3389/fpubh.2018.00149, PMID: 29942800 PMC6004510

[ref15] Buhrmester D. Furman W. Wittenberg M. T. Reis H. T. (1988). Five domains of interpersonal competence in peer relationships. J. Pers. Soc. Psychol. 55, 991–1008. doi: 10.1037/0022-3514.55.6.991, PMID: 3216292

[ref16] Burisch M. (1984). Approaches to personality inventory construction: a comparison of merits. Am. Psychol. 39, 214–227. doi: 10.1037/0003-066X.39.3.214, PMID: 9429915

[ref17] Butt A. Zahid Z. M. (2015). Effect of assertiveness skills on job burnout. Int. Lett. Soc. Human. Sci. 63, 218–224. doi: 10.18052/www.scipress.com/ILSHS.63.218

[ref18] Byrnes J. P. (2013). The nature and development of decision-making: a self-regulation model. London: Psychology Press.

[ref19] Byrnes J. P. Miller D. C. Reynolds M. (1999). Learning to make good decisions: a self-regulation perspective. Child Dev. 70, 1121–1140. doi: 10.1111/1467-8624.00082

[ref20] Carton R. B. Hofer C. W. (2006). Measuring organizational performance: metrics for entrepreneurship and strategic management research. Northampton, MA, USA: Edward Elgar Publishing.

[ref21] Ceschi A. Demerouti E. Sartori R. Weller J. (2017). Decision-making processes in the workplace: how exhaustion, lack of resources and job demands impair them and affect performance. Front. Psychol. 8:313. doi: 10.3389/fpsyg.2017.00313 28529491 PMC5418353

[ref22] Chalmers P. Pritikin J. Robitzsch A. Zoltak M. Kim K.-M. Falk C. F.. (2018). Package “mirt” (Version 1.29). Available at: https://cran.r-project.org/web/packages/mirt/mirt.pdf (Accessed February 15, 2024).

[ref23] Charoensap-Kelly P. Broussard L. Lindsly M. Troy M. (2016). Evaluation of a soft skills training program. Bus. Prof. Commun. Q. 79, 154–179. doi: 10.1177/2329490615602090

[ref24] Cheung G. W. Rensvold R. B. (2002). Evaluating goodness-of-fit indexes for testing measurement invariance. Struct. Equ. Model. 9, 233–255. doi: 10.1207/S15328007SEM0902_5

[ref25] Choi S. W. (2016). Logistic ordinal regression differential item functioning using IRT (version 0.3-3). Available at: https://cran.r-project.org/web/packages/lordif/lordif.pdf (Accessed February 15, 2024).

[ref26] Ciappei C. Cinque M. (2014). Soft skills per il governo dell’agire. Milano: Franco Angeli.

[ref27] Cimatti B. (2016). Definition, development, assessment of soft skills and their role for the quality of organizations and enterprises. Int. J. Qual. Res. 10, 97–130. doi: 10.18421/IJQR10.01-05

[ref28] Colledani D. (2018). Psychometric properties and gender invariance for the Dickman impulsivity inventory. Test. Psychomet. Methodol. Appl. Psychol. 25, 49–61. doi: 10.4473/TPM25.1.3

[ref29] Colledani D. Anselmi P. Robusto E. (2018a). Using item response theory for the development of a new short form of the Eysenck personality questionnaire-revised. Front. Psychol. 9:1834. doi: 10.3389/fpsyg.2018.01834, PMID: 30356840 PMC6190847

[ref30] Colledani D. Anselmi P. Robusto E. (2019a). Development of a new abbreviated form of the Eysenck personality questionnaire-revised with multidimensional item response theory. Personal. Individ. Differ. 149, 108–117. doi: 10.1016/j.paid.2019.05.044

[ref31] Colledani D. Anselmi P. Robusto E. (2019b). Using multidimensional item response theory to develop an abbreviated form of the Italian version of Eysenck’s IVE questionnaire. Personal. Individ. Differ. 142, 45–52. doi: 10.1016/j.paid.2019.01.032

[ref32] Colledani D. Anselmi P. Robusto E. (2024). Development of a scale for capturing psychological aspects of physical–digital integration: relationships with psychosocial functioning and facial emotion recognition. AI & Soc. 39, 1707–1719. doi: 10.1007/s00146-023-01646-9, PMID: 37358941 PMC10031718

[ref33] Colledani D. Camperio Ciani A. (2021). A worldwide internet study based on implicit association test revealed a higher prevalence of adult males’ androphilia than ever reported before. J. Sex. Med. 18, 4–16. doi: 10.1016/j.jsxm.2020.09.011, PMID: 33250358

[ref34] Colledani D. Robusto E. Anselmi P. (2018b). Development of a new abbreviated form of the junior Eysenck personality questionnaire-revised. Personal. Individ. Differ. 120, 159–165. doi: 10.1016/j.paid.2017.08.037 PMC6190847 30356840

[ref35] Comrey A. L. (1988). Factor-analytic methods of scale development in personality and clinical psychology. J. Consult. Clin. Psychol. 56, 754–761. doi: 10.1037/0022-006X.56.5.754, PMID: 3057010

[ref36] Dazzi C. Voci A. Bergamin F. Capozza D. (1998). Uno studio sull’impegno con l’organizzazione in una azienda. Bologna: Patron.

[ref37] De Carlo A. Dal Corso L. Carluccio F. Colledani D. Falco A. (2020). Positive supervisor behaviors and employee performance: the serial mediation of workplace spirituality and work engagement. Front. Psychol. 11:1834. doi: 10.3389/fpsyg.2020.01834, PMID: 32793085 PMC7393218

[ref38] De Carlo N. A. Falco A. Capozza D. (2008/2011). Test di valutazione del rischio stress lavoro-correlato nella prospettiva del benessere organizzativo, Qu-BO [Test for the assessment of work-related stress risk in the organizational well-being perspective, Qu-BO]. Milano: FrancoAngeli.

[ref39] Dehghan A. Ma’toufi A. R. (2016). The relationship between communication skills and organizational commitment to employees’ job performance: evidence from Iran. Int. Res. J. Manag. Sci. 4, 102–115.

[ref40] Del Missier F. Mäntylä T. De Bruin W. B. (2012). Decision-making competence, executive functioning, and general cognitive abilities. J. Behav. Decis. Mak. 25, 331–351. doi: 10.1002/bdm.731, PMID: 38917554

[ref41] Ellis B. H. Miller K. I. (1993). The role of assertiveness, personal control, and participation in the prediction of nurse burnout. J. Organ. Behav. 21, 327–342. doi: 10.1080/00909889309365377

[ref42] Emold C. Schneider N. Meller I. Yagil Y. (2011). Communication skills, working environment and burnout among oncology nurses. Eur. J. Oncol. Nurs. 15, 358–363. doi: 10.1016/j.ejon.2010.08.001 20863757

[ref43] Engelbrecht A. S. Heine G. Mahembe B. (2017). Integrity, ethical leadership, trust and work engagement. Leadership Organ. Dev. J. 38, 368–379. doi: 10.1108/LODJ-11-2015-0237, PMID: 39104746

[ref44] Epskamp S. Borsboom D. Fried E. I. (2018). Estimating psychological networks and their accuracy: A tutorial paper. Behav. Res. Methods 50, 195–212. doi: 10.3758/s13428-017-0862-1, PMID: 28342071 PMC5809547

[ref45] Epskamp S. Fried E. I. (2018). A tutorial on regularized partial correlation networks. Psychol. Methods 23, 617–634. doi: 10.1037/met0000167, PMID: 29595293

[ref46] Eshet Y. (2004). Digital literacy: a conceptual framework for survival skills in the digital era. J. Educ. Multimedia Hypermedia 13, 93–106.

[ref47] Fagnani M. Devita M. Colledani D. Anselmi P. Sergi G. Mapelli D.. (2021). Religious assessment in Italian older adults: psychometric properties of the Francis scale of attitude toward Christianity and the behavioral religiosity scale. Exp. Aging Res. 47, 478–493. doi: 10.1080/0361073X.2021.1913938, PMID: 33847233

[ref48] Ferguson C. J. (2016). An effect size primer: a guide for clinicians and researchers. Prof. Psychol. Res. Pract. 40, 532–538. doi: 10.1037/a0015808

[ref49] Fisher G. G. Matthews R. A. Gibbons A. M. (2016). Developing and investigating the use of single-item measures in organizational research. J. Occup. Health Psychol. 21, 3–23. doi: 10.1037/a0039139, PMID: 25894198

[ref50] Foygel R. Drton M. (2010). Extended Bayesian information criteria for Gaussian graphical models. Adv. Neural Inf. Proces. Syst. 23, 604–612.

[ref51] Friedman J. Hastie T. Tibshirani R. (2008). Sparse inverse covariance estimation with the graphical lasso. Biostatistics 9, 432–441. doi: 10.1093/biostatistics/kxm045, PMID: 18079126 PMC3019769

[ref52] Gibb S. (2014). Soft skills assessment: theory development and the research agenda. Int. J. Lifelong Educ. 33, 455–471. doi: 10.1080/02601370.2013.867546

[ref53] Guadagnoli E. Velicer W. F. (1988). Relation of sample size to the stability of component patterns. Psychol. Bull. 103, 265–275. doi: 10.1037/0033-2909.103.2.265, PMID: 3363047

[ref54] Harris M. M. Schaubroeck J. (1988). A meta-analysis of self-supervisor, self-peer, and peer-supervisor ratings. Pers. Psychol. 41, 43–62. doi: 10.1111/j.1744-6570.1988.tb00631.x

[ref55] Harvey R. J. Billings R. S. Nilan K. J. (1985). Confirmatory factor analysis of the job diagnostic survey: good news and bad news. J. Appl. Psychol. 70, 461–468. doi: 10.1037/0021-9010.70.3.461

[ref56] Hecht M. L. (1978). The conceptualization and measurement of interpersonal communication satisfaction. Hum. Commun. Res. 4, 253–264. doi: 10.1111/j.1468-2958.1978.tb00614.x, PMID: 39111216

[ref57] Henry O. (2009). Organisational conflict and its effects on organizational performance. Res. J. Bus. Manag. 3, 16–24. doi: 10.3923/rjbm.2009.16.24

[ref58] Hinkin T. R. (1995). A review of scale development practices in the study of organizations. J. Manag. 21, 967–988. doi: 10.1016/0149-2063(95)90050-0

[ref59] Hinkin T. R. (1998). A brief tutorial on the development of measures for use in survey questionnaires. Organ. Res. Methods 1, 104–121. doi: 10.1177/109442819800100106, PMID: 35601590

[ref60] Hinkin T. R. Schriesheim C. A. (1989). Development and application of new scales to measure the French and raven (1959) bases of social power. J. Appl. Psychol. 74, 561–567. doi: 10.1037/0021-9010.74.4.561

[ref61] Hogan R. Chamorro-Premuzic T. Kaiser R. B. (2013). Employability and career success: bridging the gap between theory and reality. Ind. Organ. Psychol. 6, 3–16. doi: 10.1111/iops.12001

[ref62] Hurrell S. A. (2016). Rethinking the soft skills deficit blame game: employers, skills withdrawal and the reporting of soft skills gaps. Hum. Relat. 69, 605–628. doi: 10.1177/0018726715591636

[ref63] Ibrahim R. Boerhannoeddin A. Bakare K. K. (2017). The effect of soft skills and training methodology on employee performance. Eur. J. Train. Dev. 41, 388–406. doi: 10.1108/EJTD-08-2016-0066, PMID: 39352901

[ref64] Inwald R. E. Hurwitz H. Jr. Kaufman J. C. (1991). Uncertainty reduction in retail and public safety-private security screening. Forensic Rep. 4, 171–212.

[ref65] Jackson D. Bridgstock R. (2018). Evidencing student success in the contemporary world-of-work: renewing our thinking. High. Educ. Res. Dev. 37, 984–998. doi: 10.1080/07294360.2018.1469603

[ref66] Jodoin M. G. Gierl M. J. (2001). Evaluating type I error and power rates using an effect size measure with the logistic regression procedure for DIF detection. Appl. Meas. Educ. 14, 329–349. doi: 10.1207/S15324818AME1404_2, PMID: 18774693

[ref67] Juhász T. Horváth-Csikós G. Gáspár T. (2023). Gap analysis of future employee and employer on soft skills. Hum. Syst. Manag. 42, 527–542. doi: 10.3233/HSM-220161

[ref68] Keerativutisest V. Hanson B. J. (2017). Developing high performance teams (HPT) through employee motivation, interpersonal communication skills, and entrepreneurial mindset using organization development interventions (ODI): a study of selected engineering service companies in Thailand. ABAC ODI J. Vis. Act. Outcome 4, 29–56.

[ref69] Khaouja I. Mezzour G. Carley K. M. Kassou I. (2019). Building a soft skill taxonomy from job openings. Soc. Netw. Anal. Min. 9, 1–19. doi: 10.1007/s13278-019-0583-9

[ref70] Kumar K. Beyerlein M. (1991). Construction and validation of an instrument for measuring ingratiatory behaviors in organizational settings. J. Appl. Psychol. 76, 619–627. doi: 10.1037/0021-9010.76.5.619

[ref71] Kyllonen P. C. (2013). Soft skills for the workplace. Change Mag. Higher Learn. 45, 16–23. doi: 10.1080/00091383.2013.841516, PMID: 39225839

[ref72] Lalor J. P. Wu H. Yu H. (2016). Building an evaluation scale using item response theory. Proceedings of the 2016 conference on empirical methods in natural language processing, Austin, Texas. 10.18653/v1/d16-1062 PMC5167538 28004039

[ref73] Luther N. (2000). Integrity testing and job performance within high performance work teams: a short note. J. Bus. Psychol. 15, 19–25. doi: 10.1023/A:1007762717488

[ref74] Mann L. Burnett P. Radford M. Ford S. (1997). The Melbourne decision-making questionnaire: an instrument for measuring patterns for coping with decisional conflict. J. Behav. Decis. Mak. 10, 1–19. doi: 10.1002/(SICI)1099-0771(199703)10:1<1::AID-BDM242>3.0.CO;2-X

[ref75] Marsh H. W. Hau K. T. Wen Z. (2004). In search of golden rules: comment on hypothesis-testing approaches to setting cutoff values for fit indexes and dangers in overgeneralizing Hu and Bentler’s (1999) findings. Struct. Equ. Model. 11, 320–341. doi: 10.1207/s15328007sem1103_2

[ref77] McDonnell A. (2011). Still fighting the “war for talent”? Bridging the science versus practice gap. J. Bus. Psychol. 26, 169–173. doi: 10.1007/s10869-011-9220-y

[ref78] Murugan D. M. S. Sujatha T. (2020). A study on soft skill and its impact of growth and productivity in service industry. J. Compos. Theory 13, 1–12. doi: 10.2139/ssrn.3969590

[ref79] Muthén L. K. Muthén B. O. (2012). Mplus user’s guide. 7th Edn. Los Angeles, CA, USA: Muthén & Muthén.

[ref80] Nadim M. Chaudhry M. S. Kalyar M. N. Riaz T. (2012). Effects of motivational factors on teachers' job satisfaction: a study on public sector degree colleges of Punjab, Pakistan. J. Commerce 4, 25–32.

[ref81] Nickson D. Warhurst C. Commander J. Hurrell S. A. Cullen A. M. (2012). Soft skills and employability: evidence from UK retail. Econ. Ind. Democr. 33, 65–84. doi: 10.1177/0143831X11427589

[ref82] Nisha S. M. Rajasekaran V. (2018). Employability skills: A review. IUP J. Soft Skills 12, 29–37.

[ref83] Nugraha I. G. B. S. M. Sitiari N. W. Yasa P. N. S. (2021). Mediation effect of work motivation on relationship of soft skill and hard skill on employee performance in Denpasar Marthalia skincare clinical. Jurnal Ekonomi Bisnis JAGADITHA 8, 136–145. doi: 10.22225/jj.8.2.2021.136-145

[ref84] Nusrat M. Sultana N. (2019). Soft skills for sustainable employment of business graduates of Bangladesh. Higher Educ. Skills Work-Based Learn. 9, 264–278. doi: 10.1108/HESWBL-01-2018-0002

[ref85] Ones D. S. Viswesvaran C. Schmidt F. L. (1993). Comprehensive meta-analysis of integrity test validities: findings and implications for personnel selection and theories of job performance. J. Appl. Psychol. 78, 679–703. doi: 10.1037/0021-9010.78.4.679

[ref86] Orlando M. Thissen D. (2000). Likelihood-based item-fit indices for dichotomous item response theory models. Appl. Psychol. Meas. 24, 50–64. doi: 10.1177/01466216000241003

[ref87] Paddi K. (2014). Perceptions of employability skills necessary to enhance human resource management graduates’ prospects of securing a relevant place in the labor market. Eur. Sci. J. 20, 129–143.

[ref9002] Paksoy M. Soyer F. Çalık F. (2017). The impact of managerial communication skills on the levels of job satisfaction and job commitment. J. Hum. Sci. 14, 642–652. doi: 10.14687/jhs.v14i1.4259

[ref88] Poláková M. Suleimanová J. H. Madzík P. Copuš L. Molnárová I. Polednová J. (2023). Soft skills and their importance in the labour market under the conditions of industry 5.0. Heliyon 9:e18670. doi: 10.1016/j.heliyon.2023.e18670, PMID: 37593611 PMC10428053

[ref89] Posthuma R. A. Maertz C. P. Jr. (2003). Relationships between integrity–related variables, work performance, and trustworthiness in English and Spanish. Int. J. Sel. Assess. 11, 102–105. doi: 10.1111/1468-2389.00231

[ref91] Raykov T. (2001). Bias of coefficient afor fixed congeneric measures with correlated errors. Appl. Psychol. Meas. 25, 69–76. doi: 10.1177/01466216010251005

[ref90] R Core Team (2018). R: a language and environment for statistical computing [computer software]. Available at: http://www.Rproject.org/ (Accessed February 15, 2024).

[ref92] Revelle W. (2024). Psych: procedures for psychological, psychometric, and personality research (version 2.4.1). Available at: https://cran.r-project.org/web/packages/psych/index.html (Accessed February 15, 2024).

[ref93] Rosa R. Madonna G. (2020). Teachers and burnout: Biodanza SRT as embodiment training in the development of emotional skills and soft skills. J. Hum. Sport Exerc. 15, S575–S585. doi: 10.14198/jhse.2020.15.Proc3.10

[ref94] Rummel R. J. (1970). Applied factor analysis. Evanston: Northwestern University Press.

[ref95] Salleh K. M. Sulaiman N. L. Talib K. N. (2010). Globalization’s impact on soft skills demand in the Malaysian workforce and organizations: what makes graduates employable. In Proceedings of the 1st UPI international conference on technical and vocational education and training (pp. 10–11).

[ref96] Samejima F. (1969). Estimation of latent ability using a response pattern of graded scores. Psychomet. Monogr. Suppl. 34, 1–97. doi: 10.1007/BF03372160

[ref97] Schlenker B. R. (2008). Integrity and character: implications of principled and expedient ethical ideologies. J. Soc. Clin. Psychol. 27, 1078–1125. doi: 10.1521/jscp.2008.27.10.1078

[ref98] Schmidt F. L. Hunter J. E. (1998). The validity and utility of selection methods in personnel psychology: practical and theoretical implications of 85 years of research findings. Psychol. Bull. 124, 262–274. doi: 10.1037/0033-2909.124.2.262

[ref99] Seligman M. E. P. (2002). Authentic happiness: Using the new positive psychology to realize your potential for lasting fulfilment. New York: Free Press.

[ref100] Semaan M. S. Bassil J. P. A. Salameh P. (2021). Effekte von soft skills und emotionaler Intelligenz auf burnout von Fachkräften im Gesundheitswesen: eine Querschnittsstudie aus dem Libanon [Effect of soft skills and emotional intelligence of health-care professionals on burnout: a Lebanese cross-sectional study]. Int. J. Health Professions 8, 112–124. doi: 10.2478/ijhp-2021-0011

[ref102] Sharma H. (2022). How short or long should be a questionnaire for any research? Researchers dilemma in deciding the appropriate questionnaire length. Saudi J Anaesth 16, 65–68. doi: 10.4103/sja.sja_163_21, PMID: 35261591 PMC8846243

[ref101] Sharma V. (2018). Soft skills: an employability enabler. IUP J. Soft Skills 12, 25–32.

[ref103] Soto C. J. Napolitano C. M. Sewell M. N. Yoon H. J. Roberts B. W. (2022). An integrative framework for conceptualizing and assessing social, emotional, and behavioral skills: the BESSI. J. Pers. Soc. Psychol. 123, 192–222. doi: 10.1037/pspp0000401, PMID: 35113631

[ref104] Styron K. (2023). Forward-looking practices to improve the soft skills of software engineers. Bus. Manag. Res. Appl. Cross-Disciplinary J. 2, 1–36.

[ref105] Tănase S. Manea C. Chraif M. Anţei M. Coblaş V. (2012). Assertiveness and organizational trust as predictors of mental and physical health in a Romanian oil company. Procedia Soc. Behav. Sci. 33, 1047–1051. doi: 10.1016/j.sbspro.2012.01.282

[ref106] Tassé M. J. Schalock R. L. Thissen D. Balboni G. Bersani H. Borthwick-Duffy S. A.. (2016). Development and standardization of the diagnostic adaptive behavior scale: application of item response theory to the assessment of adaptive behavior. Am. J. Intellect. Dev. Disabil. 121, 79–94. doi: 10.1352/1944-7558-121.2.79, PMID: 26914464

[ref107] Valieva F. (2020). “Soft skills vs professional burnout: the case of technical universities” in Integrating engineering education and humanities for global intercultural perspectives. IEEHGIP 2022. ed. Anikina Z., Lecture Notes in Networks and Systems, vol. 131 (Cham: Springer).

[ref109] Vandenberg R. J. Lance C. E. (2000). A review and synthesis of the measurement invariance literature: suggestions, practices, and recommendations for organizational research. Organ. Res. Methods 3, 4–70. doi: 10.1177/109442810031002

[ref108] Van Iddekinge C. H. Roth P. L. Raymark P. H. Odle-Dusseau H. N. (2012). The criterion-related validity of integrity tests: an updated meta-analysis. J. Appl. Psychol. 97, 499–530. doi: 10.1037/a0021196, PMID: 21319880

[ref110] Velicer W. F. (1976). Determining the number of components from the matrix of partial correlations. Psychometrika 41, 321–327. doi: 10.1007/BF02293557, PMID: 32063847

[ref111] Verma A. Bedi M. (2008). Importance of soft skills in IT industry. ICFAI J. Soft Skills 2, 15–24.

[ref112] Weller J. A. Levin I. P. Rose J. P. Bossard E. (2012). Assessment of decision-making competence in preadolescence. J. Behav. Decis. Mak. 25, 414–426. doi: 10.1002/bdm.744

[ref113] Widad A. Abdellah G. (2022). Strategies used to teach soft skills in undergraduate nursing education: a scoping review. J. Prof. Nurs. 42, 209–218. doi: 10.1016/j.profnurs.2022.07.010 36150863

[ref114] Yahyazadeh-Jeloudar S. Lotfi-Goodarzi F. (2012). The relationship between social intelligence and job satisfaction among MA and BA teachers. Int. J. Educ. Sci. 4, 209–213. doi: 10.1080/09751122.2012.11890044

[ref115] Yorke M. (2006). Employability in higher education: what it is-what it is not, vol. 1. York: Higher Education Academy.

[ref116] Yuan K. H. Bentler P. M. (2000). Three likelihood-based methods for mean and covariance structure analysis with nonnormal missing data. Sociol. Methodol. 30, 165–200. doi: 10.1111/0081-1750.00078

[ref117] Zanon C. Hutz C. S. Yoo H. H. Hambleton R. K. (2016). An application of item response theory to psychological test development. Psicologia Reflexão e Crítica 1, 1–10. doi: 10.1186/MSSAT1155-016-0040-x

